# Non-invasive Focal Mechanical Vibrations Delivered by Wearable Devices: An Open-Label Pilot Study in Childhood Ataxia

**DOI:** 10.3389/fneur.2018.00849

**Published:** 2018-10-09

**Authors:** Tommaso Schirinzi, Alberto Romano, Martina Favetta, Andrea Sancesario, Riccardo Burattini, Susanna Summa, Gessica Della Bella, Enrico Castelli, Enrico Bertini, Maurizio Petrarca, Gessica Vasco

**Affiliations:** ^1^Department of Neurosciences, Bambino Gesù Children's Hospital, Rome, Italy; ^2^Department of Systems Medicine, University of Roma Tor Vergata, Rome, Italy

**Keywords:** Ataxia, non-invasive stimulation, focal vibrations, equistasi, neuromodulation

## Abstract

Non-invasive focal mechanical vibrations (NIFMV) now represent a strategy of increasing interest to improve motor control in different neurological diseases. Nanotechnology allowed the creation of wearable devices transforming thermal variations into mechanical energy with focal vibrations. This kind of wearable stimulators (WS) has produced encouraging preliminary results when used in the treatment of movement disorders and ataxia in adults. In this open label pilot study we first evaluated the feasibility, safety and effectiveness of NIFMV by WS in a cohort of 10 patients with childhood ataxia, a phenomenological category including different conditions still lacking of effective symptomatic therapies. Through the assessment of both clinical rating scales and spatio-temporal gait parameters via standardized gait analysis, we observed that a 4 weeks long treatment with WS Equistasi® was safe and provided significantly different effects in stride features of patients with slow/non-progressive cerebellar ataxia and Friedreich's Ataxia. Although limited by the sample size, the absence of a placebo-controlled group, the poor compliance of enrolled population to the original experimental design and the partial accuracy of outcome measures in pediatric subjects, we suggest that NIFMV by WS could support locomotion of patients with childhood slow/non-progressive cerebellar ataxia with preserved sensory system and no signs of peripheral neuropathy. Future studies are definitely necessary to confirm these preliminary results and define criteria for successful NIFMV-based treatment

## Introduction

Childhood ataxia (CA) is a phenomenological definition labeling patients suffering with an ataxic syndrome that appeared during childhood or at least in early adolescence. The group obviously encompasses several conditions with different etiology, including genetic and acquired forms, presenting either with pure cerebellar ataxia or complex syndromes with combined sensorial and/or strength deficit, neuropathy and mental disturbances (1, 2). Although in the absence of reliable epidemiological data, a prevalence of 26/1,00,000 has been recently estimated (1). However, regardless of clinical heterogeneity, all CAs lack of effective symptomatic treatments, such that patients are burdened by poor quality of life and high socio-economic costs (3).

Non-invasive focal mechanical vibrations (NIFMV) now represent an innovative strategy to enhance balance and motor control across different neurological diseases (4). Muscle vibrations indeed activate peripheral mechanoreceptors, leading to both short-term and long-term dynamic changes within somatosensory and motor systems, such that repeated applications may promote neuroplasticity with subsequent improvement in motor behavior (4–6).

NIFMV is usually delivered through electromechanical-based devices (6); however, a novel wearable tool has been lately introduced, which is nanotechnology-based, transforming minimal thermal variations into mechanical energy by self-producing of a focal vibration (7).

Encouraging results have been obtained from the use of such wearable stimulators (WSs) as a support to improve locomotor abilities in patients with Parkinson's Disease (PD) (8, 9) and, of interest, also in adult patients with hereditary cerebellar ataxias (10). Conversely, no data are available yet on patients with CA. In this pilot open-label trial, we thus attempted at evaluating the feasibility, safety and effectiveness of NIFMV by WS in CA, in order to explore novel therapeutic opportunities for this incurable condition.

## Methods

### Study population

The study included 10 consecutive patients with CA (onset of ataxia <18 years of age) afferent to Bambino Gesù Children's Hospital (Rome, Italy) between 2017 and 2018. Exclusion criteria were severe motor disability (Item 4 of Scale for Assessment and Rating of Ataxia, SARA > 4) and intellectual disability (IQ < 55). The cohort encompassed 6 Friedreich's Ataxia (FRDA) patients and 4 patients with other slow/non-progressive cerebellar ataxias (OA), specifically 3 with phosphomannomutase (PMM2) deficiency, 1 with isolated cerebellar atrophy. They all received brain MRI to exclude secondary causes of ataxia and nerve conduction study/electromyography to screen the presence of neuropathy. Diagnosis was obtained by appropriate genetic tests.

### Intervention

**Figure 1:**
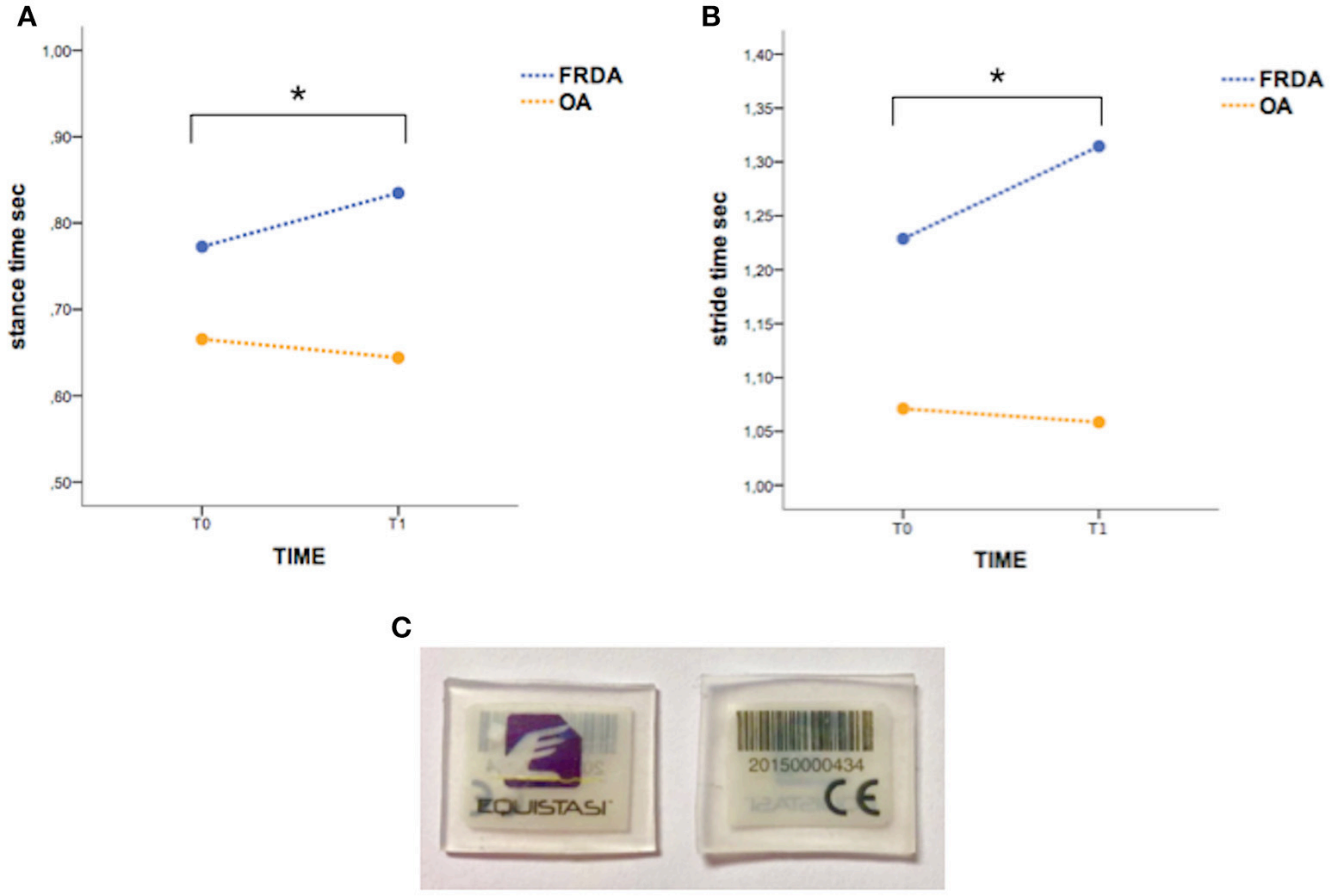
The “stance time” **(A)** and “stride time” **(B)** variations in T0-T1 interval are significantly different between FRDA and OA. Asterisks indicate statistical significance (*p* < 0.05). **(C)** The picture shows WS Equistasi® devices.

The WS Equistasi® (Equistasi S.r.l., Milan, Italy) consists of a rectangular plate (10 × 20 × 0.5 mm, 0.17 gr), composed by nanotechnology fibers which produces, at body temperature, mechanical vibrations with a frequency of about 9000 Hz and a very low pressure of about 3-4 E-6 Pa. Equistasi® is a registered medical device (class 1, ministerial code n. 342577 on 05/08/2010), safe for humans (7) (Figure 1C).

### Trial design and outcome measures

All patients, after offering their consent (or parental consent, when minors), received a baseline assessment (T0). Participants were invited to wear three WSs, one over the seventh cervical vertebra and one on each soleus muscle tendons as previously described (8, 10), by using commercial patch/plasters. Application was prescribed for 60 min/day for 5 days in the first week, and then for 120 min/day (1 h in the morning, 1 h in the afternoon), 5 days/week for the following 3 weeks. At the end of the intervention, a second assessment was planned (T1). Then, a 4 weeks long period of WS wash-out was imposed, followed by the final assessment (T2). All patients were subject to the same protocol of physiotherapy (50 min individual session for gait and balance training, 3 times/week) over the entire trial. Also individual medical therapy continued unchanged.

Efficacy of WS was measured by means of the evaluation of clinical changes across the different time-points. Assessment performed at T0, T1, and T2 included full medical examination, clinical evaluation by SARA, 9 holes peg test (9-HPT) for both dominant and non-dominant hand, 6 min walking test (6MWT). Because of the age-related reliability of clinical rating scales (11, 12), also spatio-temporal gait parameters were considered as outcome measures to test WS effectiveness. Standardized gait analysis was conducted by an optoelectronic motion capture system with eight-camera (Vicon MX, UK) at the sampling rate of 200 Hz, as previously described (12–15). Subjects received 33 markers located on anatomical landmarks as indicated by the Plug-in-Gait protocol in order to reconstruct a full body kinematic and kinetic model. Collected data were normalized according to anthropometric features (12, 13). The following spatio-temporal parameters were specifically considered for this study: foot off (% gait cycle), stride width (meters), stance time (seconds), stride velocity (meters/second), stride time (seconds), swing time (seconds), stride length (meters), step length (meters), double support (seconds). For each variable, average values of three significant trials were analyzed. Given the non-variability between the two body sides, data from both lower limbs were analyzed together.

Safety of WS was monitored at every visit. E-mail and phone contacts were provided for adverse events communication.

The study was conducted in the context of the protocol code 1166/2016, approved by the local ethic committee (Bambino Gesù Children's Hospital). The intervention and all the procedures here performed were in agreement with the local ethical standards and the ethical principles of Helsinki declaration.

### Statistical analysis

Distribution of all collected variables was preliminary examined by the Shapiro-Wilk test. Differences in demographic, clinical and gait parameters between FRDA and OA groups were analyzed with parametric (one-way ANOVA) or non-parametric (U-Mann-Whitney) tests, as appropriate. To measure changes in clinical and gait parameters, the repeated measures ANOVA with TIME (T0, T1, T2) and GROUP (FRDA, OA) as within-subject factors was performed, by using the Greenhouse-Geisser correction for non-spherical data (Mauchley's test examined for sphericity). Statistical significance was set at *p* < 0.05. Analysis was conducted by IBM-SPSS software.

## Results

Demographic, clinical and gait features of study population are summarized in Table 1. No significant differences emerged between FRDA and OA groups in age, gender distribution and body mass index (BMI). FRDA patients all had peripheral neuropathy and sensory impairment; none of OA patients showed neither clinical nor instrumental findings of peripheral nerves impairment and sensory deficit.

**Table 1:** Summarizes main demographic, clinical and gait features of the whole study population and both FRDA and OA subgroups at T0 and T1 time-points.

	**T0**			**T1**		
	**FRDA**	**OA**	**All**	**FRDA**	**OA**	**All**
**N**	6	4	10	–	–	–
**Age (y)**						
mean	15.66	10.00	13.10	–	–	–
st.dev.	8.40	4.80	7.30	–	–	–
**Gender**						
(M/F)	2/4	3/3	5/6	–	–	–
**BMI**						
mean	18.40	15.98	17.32	–	–	–
st.dev.	4.05	2.72	3.58	–	–	–
**SARA**						
mean	13.83	12.13	13.15	11.70	11.75	11.72
st.dev.	6.53	5.54	5.89	5.07	6.76	5.48
**9-HPT dominant (s)**						
mean	44.82	43.67	44.36	38.04	45.16	41.20
st.dev.	14.45	15.96	14.19	7.34	13.57	10.49
**9-HPT non dominant (s)**						
mean	50.69	53.24	51.71	47.26	58.93	52.44
st.dev.	18.11	21.37	18.34	14.37	20.85	17.44
**6MWT (m)**						
mean	349.96	305.01	334.98	356.00	372.10	363.16
st.dev.	99.62	155.34	106.39	168.75	131.63	144.25
**Foot off (%gc)**						
mean	62.04	61.90	61.99	62.91	60.58	62.03
st.dev.	3.95	2.17	3.20	2.89	2.69	2.88
**Stride width (m)**						
mean	0.22	0.20	0.21	0.22	0.19	0.21
st.dev.	0.05	0.02	0.04	0.07	0.03	0.05
**Stance time (s)**						
mean	0.77	0.67	0.73	0.83	0.64	0.76
st.dev.	0.26	0.10	0.21	0.23	0.13	0.21
**Stride velocity (m/s)**						
mean	0.89	0.94	0.91	0.79	0.95	0.85
st.dev.	0.29	0.13	0.24	0.17	0.13	0.17
**Stride time (s)**						
mean	1.23	1.07	1.17	1.31	1.06	1.22
st.dev.	0.32	0.14	0.27	0.30	0.16	0.28
**Swing time (s)**						
mean	0.46	0.41	0.44	0.48	0.41	0.46
st.dev.	0.07	0.04	0.06	0.07	0.04	0.07
**Stride length (m)**						
mean	1.02	1.01	1.02	0.99	1.02	1.00
st.dev.	0.15	0.27	0.18	0.06	0.28	0.16
**Step length (m)**						
mean	0.51	0.51	0.51	0.50	0.51	0.50
st.dev.	0.08	0.14	0.10	0.04	0.13	0.08
**Double support (s)**						
mean	0.17	0.12	0.15	0.17	0.11	0.15
st.dev.	0.10	0.03	0.79	0.09	0.04	0.08

*n, number; y, years; M, male; F, female; s, seconds; m, minutes; %gc, % gait cycle*.

All subjects used WSs as prescribed, running normal daily activities along the treatment, avoiding sedentariness during WSs application, continuing usual medical and physical therapies. No adverse events were reported. Only 4 patients concluded the study; 6 patients dropped-out at T1 because the absence of subjective clinical improvement and discomfort of WSs application. Specifically they referred not being autonomous in the application of WSs and complained about patch application (pain, itch).

The rate of drop-out at T1 was particularly high (60%); for this reason, the statistical analysis was conducted by using T0 and T1 as time-points in TIME factor, whereas data obtained from the 4 patients accomplishing T2 were not included in the model. Repeated measure ANOVA did not show a significant TIME effect on both clinical scores (SARA, 9-HPT for both hands, 6MWT) and gait parameters in the whole population. Conversely, a significant TIMExGROUP effect resulted in either “stance time” [*F*_(1, 6)_ = 7.82, *p* < 0.05] (Figure 1A) or “stride time” [*F*_(1, 6)_ = 5.54, *p* < 0.05] (Figure 1B), suggesting that the clinical condition might affect the treatment outcome. Indeed, although in the absence of statistical significance, the gait parameters and the clinical scores (SARA, 6MWT) slightly improved in OA group according with an increase of gait speed (Table 1).

## Discussion

This small open-label study aimed at exploring safety, feasibility and efficacy of NIFMV delivered by WS in CA. Our preliminary results show that treatment is safe and that the underlying disease may condition the outcome. In fact, while no changes have been observed in the whole cohort, differences instead emerged by means the group analysis. Specifically, the gait parameters (“stance time” and “stride time”) significantly differed between patients with slow/non-progressive cerebellar ataxia and FRDA after the treatment. Conversely, no significant effects were noticed in standard clinical scores (SARA, 9-HPT, and 6MWT).

The trial was definitely affected by several limitations. First, the small sample size, essentially due to both the rarity of the condition and inclusion/exclusion criteria of the study, may have influenced statistical significance of the results. Then, the study was not placebo-controlled. At this regard, the original experimental protocol was including a second follow up (T2), 1 month after the treatment wash-out; however, the high rate of drop-out (60% at T1) due to the poor compliance of enrolled population led us to perform a comparison between T0 and T1, excluding the few T2 data. The peculiar age of the study cohort (13.1 ± 7.3 years) influenced not only the compliance to the trial, but also the reliability of adopted outcome measures, which are typically age-dependent. Actually, SARA score is inaccurate in children < 11 years old (11). Moreover, gait analysis may have been conditioned by the inconstant collaboration of younger subjects. Nevertheless, no other age-specific ataxia outcome measures are available. All this should be thus considered in the final interpretation of the results and in the setting of future confirmative studies.

Despite these limitations, our study seems to highlight the mild beneficial effects of NIFMV by WS in gait features of a selected group of CA patients. These findings, if on the one hand, overlap with a **previous work** (10) showing the WS-induced improvement of gait ataxia in adult patients, on the other hand, provide adjunctive information on the specific action of NIFMV. In fact, we observed that NIFMV by WS did not change relevantly gait parameters in a small group of young FRDA patients, whereas they tended to be more effective in patients with pure slow/non-progressive cerebellar ataxia without peripheral neuropathy and sensory deficit, although in the absence of full statistical significance.

It has been demonstrated that the WS Equistasi® exerts neuromodulatory effects via the stimulation of proprioceptive reflexes, increasing the H-reflex inhibition and reducing alpha-motoneuron excitability, which in turn probably induces other adaptive changes within the proprioceptive pathways (7). Furthermore, NIFMV are able to modify sensory-motor cortical activity, contributing to the regulation of motor behavior at higher level (5). The integrity of sensory system is thus crucial to mediate the effects of NIFMV and determining the clinical effects. According with this data, we can refer the differences in treatment outcome between OA and FRDA groups to the impairment of sensory conduction, which is a stigmata of FRDA (16, 17). In addition, since FRDA is a neurodegenerative disease whose progression is faster and greater in patients with younger onset (18), clinical decline of this group of patients might also have contributed to different results.

Existing evidence indicates that NIFMV modulate neural transmission at different levels of CNS, contributing to motor control in deficient conditions (4). The availability of wearable devices for NIFMV, allowing easy, remote or home-based, continuative stimulations therefore should promote the use for symptomatic relief and supportive tool in neurorehabilitation of adult patients with ataxia or movement disorder (8–10).

Our preliminary findings also suggest that NIFMV by WS could represent a viable option of supportive therapy for patients with CA presenting with slow/non-progressive cerebellar ataxia in the absence of sensory involvement, although the individual compliance is fundamental for the final outcome. However, larger studies are necessary to confirm these preliminary observations, to define standardized schemes of treatment and the correct criteria of eligibility, especially in complex conditions such as CA.

## Author contributions

TS, GV, AS, and MP conceived the study, analyzed data and wrote the manuscript. AR, MF, RB, and SS provided assessment and analyzed data. EB, EC, and GD contributed to interpretation of results and edited the manuscript.

### Conflict of interest statement

The authors declare that the research was conducted in the absence of any commercial or financial relationships that could be construed as a potential conflict of interest.
